# Optimization of Methods for the Quantitative Analysis of Global Cell Surface Proteome and Cell Surface Polarization

**DOI:** 10.3390/ijms262311570

**Published:** 2025-11-28

**Authors:** Katalin Kuffa, Tamás Langó, András Czirók, Júlia Tárnoki-Zách, Szilvia Bősze, Gábor E. Tusnády, Zoltán Szabó

**Affiliations:** 1 Doctoral School of Biology, Institute of Biology, ELTE Eötvös Loránd University, Pázmány P. stny. 1/C, H-1117 Budapest, Hungary; 2 Protein Bioinformatics Research Group, Institute of Molecular Life Sciences, Research Centre for Natural Sciences, HUN-REN, Magyar Tudósok körútja 2, H-1117 Budapest, Hungary; 3 Department of Biological Physics, Eötvös Loránd University, Pázmány P. stny. 1/A, H-1117 Budapest, Hungary; 4 HUN-REN-ELTE Research Group of Peptide Chemistry, Hungarian Research Network, Eötvös Loránd University, Pázmány P. stny. 1/A, H-1117 Budapest, Hungary; 5 Department of Genetics, Cell- and Immunobiology, Faculty of Science, Semmelweis University, Nagyvárad tér 4, H-1089 Budapest, Hungary; 6 Department of Bioinformatics, Semmelweis University, Tűzoltó u. 7, H-1094 Budapest, Hungary; 7 Department of Medical Chemistry, Albert Szent-Györgyi Medical School, University of Szeged, H-6725 Szeged, Hungary

**Keywords:** cell surface, polarization, proteomics

## Abstract

The cell surface proteome of polarized epithelial cells plays a central role in barrier function, signaling, and vectorial transport, yet the quantitative characterization of their surface proteins remains technically challenging. We developed an optimized chemoproteomic strategy specifically tailored to studying the surface proteins of polarized cells while keeping membrane integrity intact. By applying a disulfide-linked membrane-impermeable biotin reagent, labeling was restricted to extracellular regions of transmembrane proteins (TMPs) and secreted proteins, thereby minimizing contributions from intracellular contaminants. Following biotinylated peptide-level or protein-level enrichment and mass spectrometric analysis, we systematically compared data-dependent (DDA) and data-independent acquisition (DIA) approaches, showing that while DIA increases proteome coverage, DDA more reliably identifies biotinylated peptides in our studies. To ensure robustness, we established replicate-based normalization and contaminant-aware quality control metrics that minimize biases from proteins in cell culture medium and damaged cells. The application of the workflow to Madin–Darby canine kidney (MDCK) II epithelial monolayers enabled the large-scale quantification of apical versus basolateral domains, yielding over 2100 proteins, with 235 showing significant polarized distribution, in agreement with known biology. This method offers high specificity for the extracellular labeling and quantitative resolution of cell surface protein (CSP) polarization, providing a powerful platform for studying epithelial biology and identifying extracellular epitopes relevant to diagnostics and therapeutic targeting.

## 1. Introduction

Epithelial cells play a crucial role in the absorption and secretion of materials, the recognition of external stimuli, and the protection of organs. Packed tightly together, they form a barrier surrounding various regions of organs, such as the respiratory tract [1], gastrointestinal tract [2], skin [3], and renal collecting duct [4]. Epithelial cell monolayers are often grown on permeable supports to create an in vitro barrier model, which is used to determine the uptake, efflux, and transepithelial transport kinetics of various compounds [5,6,7], providing potentially clinically relevant information. These cell monolayers are typically polarized and contain two different membrane domains within the same cell that are separated by tight junctions [8], where well-known TMPs such as occludin [9], claudins [10], and junctional adhesion molecules [11] maintain strong cell-to-cell contact and prevent the free diffusion of molecules, including ions, nutrients, and tissue specific targeted compounds [12,13]. The two domains are known as the apical and basolateral membrane regions, and the amount of various proteins in these regions results in vectorial transport of drugs [14]. Despite the biological importance of these epithelial layers, information on the distribution of CSPs in the different membrane domains is limited and scattered throughout the literature [15].

Existing techniques for determining CSP distribution using polarized cells can be categorized according to the number of proteins targeted per experiment (unique or multiple proteins). A common instance in the first group is when the coding sequence of the protein of interest (POI) is transfected into an appropriate cell type and its localization is determined based on antibody staining using confocal laser scanning microscopy, as with MDR1-transfected LLC cells [16]. Immunostaining can be performed in the natural tissue environment. For example, the SARS-CoV-2 receptor (also known as angiotensin-converting enzyme-2, ACE2) was detected in the apical region of alveolar type II cells [17], and ADAM metalloproteinase domain 9 (ADAM9) was localized apically in pancreatic tissues [18]. Sometimes, the examined proteins are genetically fused with reporter/fluorescent proteins, such as EYFP-fused NaPi-2b, which is expressed in microvilli on the apical membrane [19], and sodium channel subunit beta 2, which is tagged by YFP and microscopically detected in the apical membrane domain [20]. While these approaches yield highly specific data on the abundance of a single target protein within defined membrane domains, a primary limitation is that a prolonged time is required for cloning and stable transfection. Furthermore, fusion with a reporter protein can occasionally alter the localization or function of the target protein [21,22], making the results ambiguous and unreliable. Another drawback is that these methods mostly depend on available antibody collections [23].

To address these limitations, proteome-wide experimental strategies have gained prominence in recent decades. These strategies enable the simultaneous quantification of multiple protein abundances through advances in proteomics technologies [24,25]. Among high-throughput proteomics techniques, bottom-up protein analysis is the most widely applied to polarized cells. In this approach, proteins are digested into peptides by proteolysis and subsequently quantified using tandem mass spectrometry [26,27,28]. An appropriate sample preparation procedure invariably precedes these approaches. These methods are often combined with gel separation techniques, which are disadvantageous in terms of reaction time [27,29], as well as with apical and basolateral domain-selective or proximity-based biotinylation. This facilitates the spatial proteomic analysis of epithelial cells. The most frequently used warheads of biotinylation agents are N-hydroxysuccinimide (NHS) [29], hydrazide [25], or phenol [27]. These can react with deprotonated amino groups, such as lysine side chains [30], oxidized glycans of glycosylated proteins (as used in Cell Surface Protein Atlas [31]), or tyrosine [27,32], respectively. For example, MDCKII cells were cultured in the presence of light or heavy isotopic residues in separate plates, followed by domain-selective biotinylation of proteins using an NHS-ester-containing reagent. The labeled proteins were then captured on avidin resin, and the apical–basolateral distribution of proteins was subsequently determined from the isotope ratios [29]. Although isotope labeling strategies are indispensable for absolute quantification, they typically require specialized laboratory facilities and are costly [33]. To circumvent these limitations, label-free quantification (LFQ) is frequently employed for the analysis of polarized cells [28,34,35]. Automated DDA has been the method of choice in such studies; however, DIA has recently become available for bottom-up proteomics [36], offering more comprehensive protein identification and quantification in complex samples. However, the analysis of DIA data relies on spectral libraries, and until recently, had limited applicability for identifying chemically modified peptides.

The PolarProtDb is the most comprehensive database containing experimentally verified apical and basolateral localization of mammalian proteins [15]. Its data was derived from the above-mentioned assays, making it a valuable benchmark for evaluating the results of newly developed methods and protocols, including those of the present study.

The aim of this study was to develop a fully optimized Sulfo-NHS-SS-biotin labeling method combined with LFQ-based tandem mass spectrometry to determine the apical and basolateral distribution of several hundred proteins in polarized MDCKII cells, as well as the CSP abundance. This approach sought to overcome the aforementioned methodological limitations while minimizing protein contaminants by using control isolates. In brief, MDCKII growth time on transwell inserts was optimized using transepithelial electrical resistance (TEER) measurements. This reduced the transfer of the biotinylation reagent to the opposite side during labeling, thereby preventing inappropriate protein modification. MDCKII monolayers were characterized using both labeled peptide-level and labeled protein-level enrichment strategies; the former facilitated the identification of modified sites [30], while the latter yielded improved peptide coverage and consequently more reliable protein quantification. DDA and DIA methods were applied to all samples to ensure more comprehensive profiling of the epithelial proteins. Finally, unlabeled and labeled control samples were analyzed to identify contaminant proteins, most of which likely originated from fetal bovine serum (FBS), a common component of culture media [37].

## 2. Results

### 2.1. Identification of Cell Culture Medium Contaminants

Cell culture medium without target cells was analyzed before and after peptide-level enrichment to identify all possible proteins and biotin-labeled peptides. These contaminants are expected to be present entirely in the extracellular space and be labeled and enriched with high efficiency. A total of 290 bovine proteins were identified in these samples (see Supplementary Table S1), and these sequences were added to the canine sequence database as contaminants for all subsequent analyses. In addition to the total level of contaminants in the samples, the labeling efficiency of the completely extracellular medium proteins may also serve as a quality control measure; therefore, we further investigated the data at the peptide level. We identified biotinylated peptides in 42% and N-terminal labeling in 20% of these proteins.

### 2.2. Selection of Most Effective Enrichment and Data Analysis Methods

To compare protein-level and peptide-level enrichment methods, two replicates of cell cultures were labeled on the apical and basolateral sides and analyzed using different methods. For the peptide-level enrichment method, the labeled cell lysates were digested using an in-solution method that did not involve reducing disulfide bridges or alkylating cysteines. This was performed to prevent the loss of biotin labels, which are attached to the linker via a disulfide bridge. The enriched peptides were eluted from the neutravidin resin by reducing this disulfide bond. This step was followed by alkylation of the free sulfhydryl groups. For protein-level enrichment, intact proteins were bound to the resin and eluted in a similar way. To digest the eluted proteins, on-pellet- and on-filter-aided protocols were evaluated. LC-MS measurements of all the above samples were performed using both DDA and DIA methods. The DDA data were analyzed in Fragpipe using the Uniprot dog proteome, to which the previously identified bovine serum proteins, porcine trypsin, streptavidin, and neutravidin were added.

DIA data were analyzed in DIA-NN using three different spectral libraries: (1) created from experimental spectra of DDA measurements, (2) created by spectrum prediction for peptide sequences of proteins identified in DDA measurements, and (3) created by spectrum prediction for peptides of proteins in the entire canine proteome sequence database used for DDA analysis.

**Figure 1:**
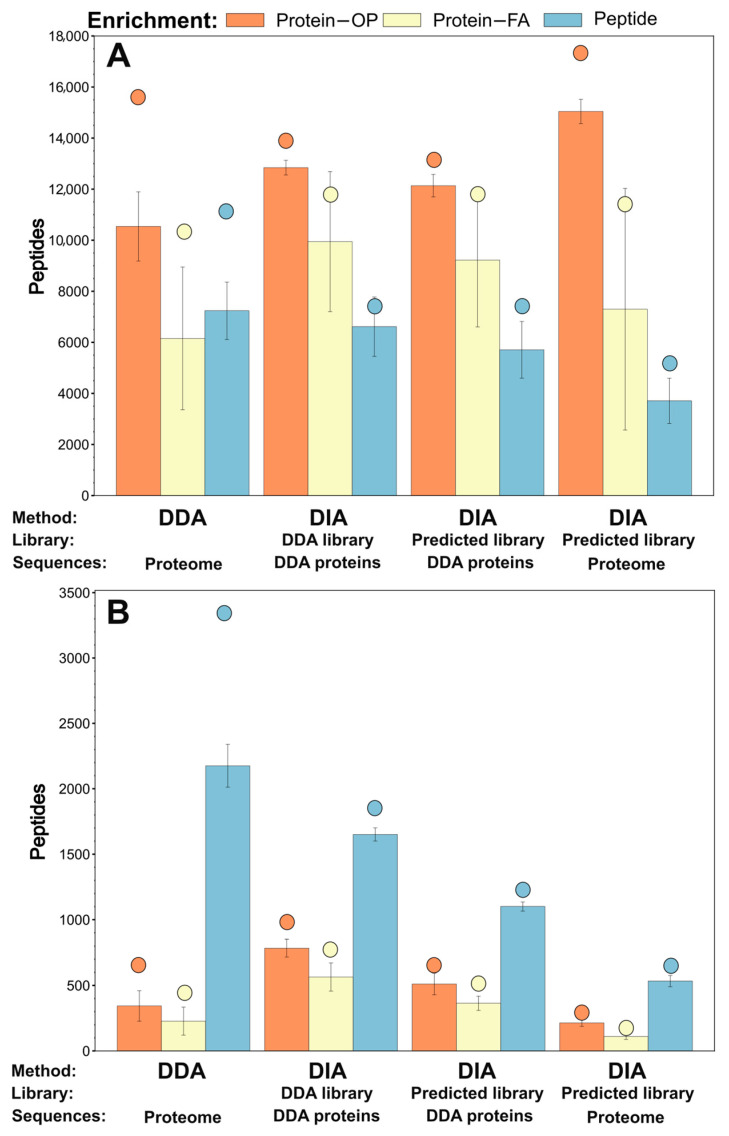
Comparison of different enrichment methods: biotinylated protein enrichment followed by on-pellet digestion (Protein–OP); biotinylated protein enrichment followed by filter-assisted digestion (Protein–FA); and biotinylated peptide enrichment (Peptide) after in-solution digestion. The number of all identified peptides (**A**) and biotinylated peptides (**B**) is shown using different MS measurement methods (DDA or DIA) and different spectral libraries for DIA data (DDA-based experimental library; a small predicted library based on protein sequences identified in DDA measurements; and a large predicted library based on the whole Uniprot dog proteome). Bars represent the average (+/− SD) number of identified peptides and the colored dots above the bars represent the total number of peptides identified in all samples.

Figure 1 shows the total number of peptides identified (Panel A) and the number of biotinylated peptides identified (Panel B) using the different methods. Protein-level enrichment using on-pellet digestion and DIA analysis with a predicted library of the whole proteome gave the highest number of peptide identifications. The total number of peptides identified in protein-enriched samples digested on-filter was almost independent of the analysis method. However, this digestion protocol provided fewer and less reproducible identifications than the on-pellet approach. In contrast, the highest number of biotinylated peptide identifications was obtained when peptide-level enriched samples were analyzed using the DDA method. Although the DDA method yields the highest number of total identifications in all samples, it also produces a larger fraction of missing values (an average of 2175 were identified in a single sample out of 3375 total biotinylated peptides). Therefore, the well-known benefit of DIA of a decreased number of missing values (although match between runs (MBR) was enabled in both methods), is balanced by the loss of information on labeling, which could confirm the extracellular presence of the analyzed proteins. DIA was more effective in identifying modified peptides using the smaller libraries; the best performance was achieved with the DDA-based experimental spectral library (Figure 1B).

**Figure 2:**
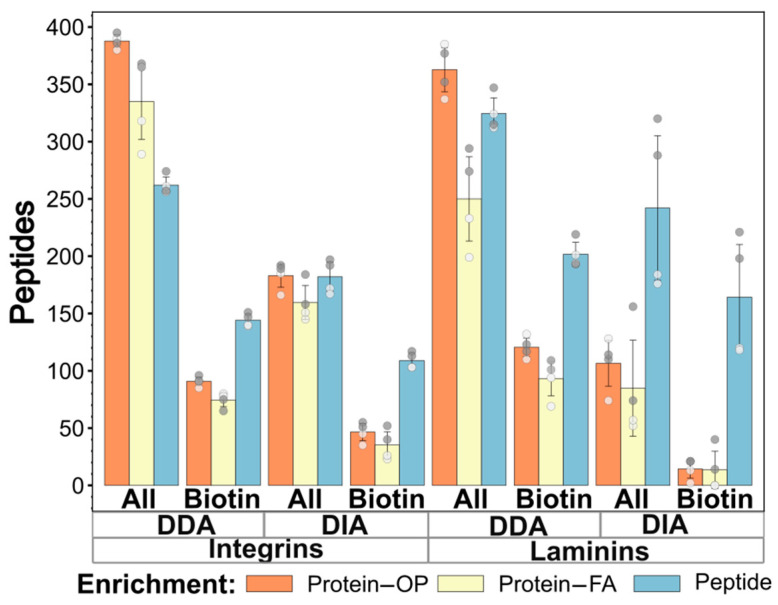
Comparison of the efficiency of different enrichment and MS measurement methods (DDA and DIA with DDA-based library) for identifying the selected extracellular cell surface markers, integrin and laminin peptides. The bars represent the average of (+/− SD) summed number of all identified (columns All) and biotinylated peptides (columns Biotin) assigned to various integrin and laminin genes (ITGA1/2/3/5/6, ITGAV, ITGB1/3/4/5/6/8, LAMA3, LAMB1/3, LAMC1/2). Dark and light dots represent the data from individual basolateral and apical samples, respectively.

To make further comparisons, we searched for marker proteins or groups of proteins that could be used for quality control and protocol optimization in cell surface proteome and polarization analysis, using the applied labeling methods. Based on the number of biotinylated peptides in topologically correct positions and the literature data, we chose integrins and laminins, which mainly localize to the extracellular side of the plasma membrane and are heterodimers or heterotrimers of different subunit polypeptide chains. Figure 2 summarizes the number of total and biotinylated peptides assigned to the 12 integrin and 5 laminin subunits identified in the samples. On-pellet digestion provides the highest number of peptide identifications for protein enrichment, with a slight difference compared to peptide enrichment for laminins. A large number of biotin-labeled peptides were identified in these proteins, which were correctly localized not only in the extracellular laminins, but also in the transmembrane integrins with known topology. Therefore, these protein groups appear to be suitable markers for cell surface labeling efficiency. For analyzing these biotinylated peptides with confirmed topology, peptide enrichment was most effective when combined with DDA analysis. Figure 2 only presents the results of DIA analysis using a DDA-based library, as this method was found to be the most effective for identifying labeled peptides from DIA data (as shown in Figure 1). The variance in identification numbers is caused by the polarization of some of these proteins, resulting in more identifications on the basolateral cellular side.

### 2.3. Differential Analysis of Apical and Basolateral Sides

Based on the data described in the previous section, we chose the peptide enrichment method using DDA analysis to perform a differential comparison of the apical and basolateral sides of MDCKII cultures, with a larger number of replicates. In total, seven apical and seven basolateral samples were produced and analyzed in three batches. Pairs of cell cultures were grown and labeled on the apical or basolateral side in parallel under identical conditions and using the same batches of reagents; therefore, members of one pair were considered one bioreplicate.

**Figure 3:**
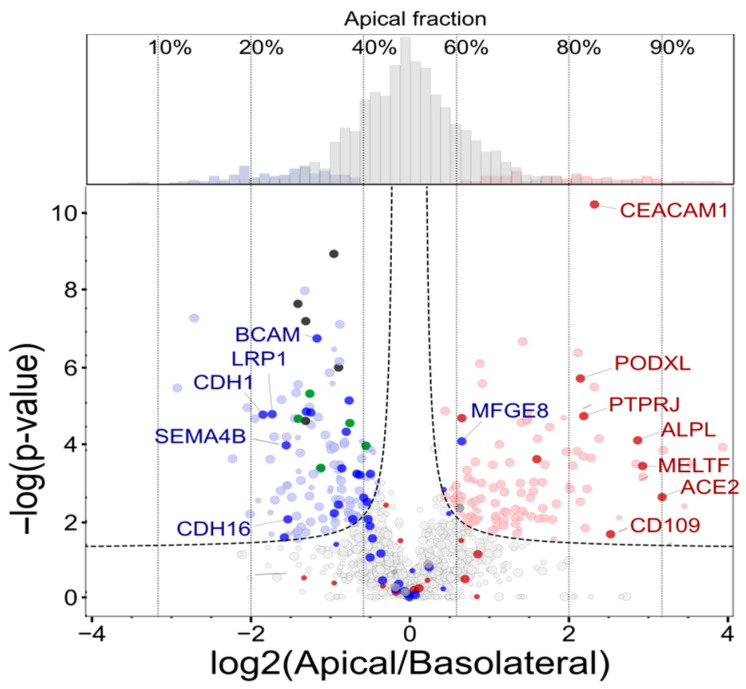
Volcano plot (−log(p) vs. log2(Fold change) of the differential analysis of the apical and basolateral sides of MDCKII cells. The distribution of percent polarization values (% apical fraction) for specific fold change values is shown at the top. The statistical significance threshold (FDR < 0.05) is shown by the dashed line. Proteins that are significantly apical or basolateral polarized are colored light red or light blue, respectively. Proteins with at least one identified biotin label are shown as large dots. Proteins that can be found in PolarProtDb are colored dark red or blue based on their apical or basolateral assignment in the database. Selected highly polarized proteins are labeled with gene names. Significantly polarized integrins and laminins are colored green and black, respectively.

The initial statistical analysis of the absolute MS intensities of the biotinylated peptides revealed a certain degree of batch effect. Therefore, we introduced bioreplicate-based intensity normalization to provide statistical power for quantifying differences in protein intensity measured on both sides of cell cultures. We chose a method that considers replicate information, resulting in normalized data that remains on the same scale and can therefore be statistically evaluated using the same methods as the measured MS intensities. This was achieved by setting the average intensity of each protein in each sample to the overall average of all samples. In each bioreplicate, this average was multiplied by a factor representing the ratio of the specific sample (cell side) to the average of the two sides in the same replicate (See Equation (1) in Section 4.6). Another advantage of this protein level normalization is that the normalization factors are unaffected by other proteins; therefore, the variable level of contaminants will not introduce bias, as it would with methods based on total or median sample intensity. This method was compared to two other batch correction methods (limma and ComBat). Supplementary Figure S4 shows that our method performed similarly in decreasing the coefficient of variation as the ComBat method (median around 20%); however, as the Apical/Basolateral ratios in individual replicates were not altered, nearly 20% more significant differences (230 vs. 193) could be detected using our method of normalization and the same statistics (FDR < 0.05). Therefore, the replicate average-based normalization was used in the further statistical analysis. Differential analysis was performed on both peptide and calculated protein intensities, which were provided by the Ionquant component of the Fragpipe DDA analysis software. Further analysis and discussion are based on quantitative protein data. However, the number of correctly biotinylated peptides, the number of peptides that changed significantly, and the median peptide apical polarization were used to validate the polarization calculated at the protein level (see Supplementary Table S2). The benefits of our normalization method are clearly evident in the differential expression analysis results comparing the apical and basolateral sides. Using traditional median-based sample normalization for peptide-enriched samples, only 21 peptides were found to have significantly different levels (FDR < 0.05) on the two cell sides. However, after bioreplicate-based normalization, 976 peptides were found to be significantly different using the same statistical approach and limits. Supplementary Figure S1 shows peptide- and protein-level volcano plots, highlighting peptides of the well-known apical marker Podocalyxin (PODXL) and basolateral marker ATPase Na+/K+ Transporting Subunit Beta 1 (ATP1B1), demonstrating peptide-level confirmation of protein-level observations. Figure 3 shows a volcano plot of the protein-level data, highlighting the proteins found in PolarProtDb. The cellular polarization of all the proteins that were found to be significantly different were in accordance with the database assignment, except for one protein (MFGE8), which is categorized as basolateral in PolarProtDb but has slightly (62%) and significantly higher apical polarization in our dataset. Many proteins expected to be polarized according to the database were not significantly different on the two sides in our data; however, they mostly showed tendencies that agreed with the expectation. The validity of our quantitative analysis of the normalized data is confirmed by the symmetrical distribution of polarization data (see histogram at the top of Figure 3). The sensitivity of this method is demonstrated by the minimal fold changes estimated based on sample randomization. According to this, as little as 55–45% polarization can be considered statistically significant at an FDR < 0.05. However, we believe that polarization of at least 60–40% can be considered biologically significant. Thus, we applied this additional biological significance threshold in subsequent analysis and discussion. Summarizing these results, from 14 samples we identified 2106 proteins; from these, 1364 and 742 proteins were expressed dominantly in the apical and in the basolateral domain, respectively. From this list, 235 proteins were significantly and quantitatively shifted to the apical or basolateral membrane direction (based on protein-level DE analysis). In the Section 3, proteins with statistically and biologically significant polarization were considered part of the core polarized proteome (235 proteins) and were used as a reference in the protein covariance analysis.

### 2.4. Covariance Analysis to Identify Protein Groups Showing Similar Polarization

**Figure 4:**
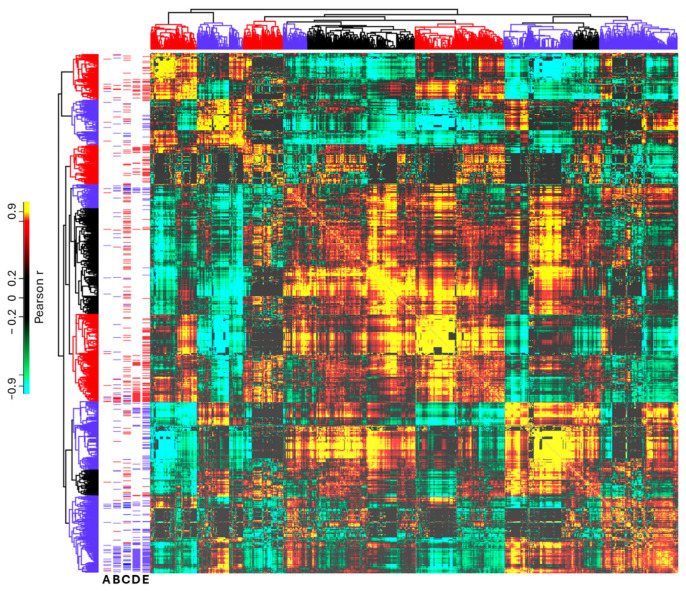
Heatmap of protein covariance analysis. Hierarchical cluster analysis was performed on protein–protein correlation coefficients. Protein polarization markers are shown on the left, according to PolarProtDb (A), Koetemann et al. [38] (B), Caceres et al. [29] (C), statistical significance (FDR < 0.05) (D), and biologically meaningful polarization (<40%: basolateral, >60%, apical, no statistical criteria) (E). The polarization markers and clusters are colored blue (basolateral) or red (apical).

Next, a covariance analysis was performed on the identified proteins to highlight clusters of proteins showing similar variations among samples. As the main factor affecting protein abundance is the analyzed cellular side, we expected to identify protein groups showing similar polarization between apical and basolateral sides. Pearson correlation coefficients were calculated for each protein pair, and then proteins were filtered to include only those with at least one strong correlation (r > 0.95). Hierarchical clustering was performed on the remaining 1481 proteins (1397 proteins without contaminant proteins), and clusters were identified in which proteins of the previously defined core polarized proteome were enriched (Fisher’s exact test with a Benjamini–Hochberg FDR < 0.05) and the intracluster average correlation coefficient was at least 0.9. This identified three apical and four basolateral clusters, comprising 450 proteins. Figure 4 shows a heatmap of these clusters and their agreement with the core proteome and the literature data. A protein–protein correlation matrix can be found in Supplementary Table S4.

### 2.5. Comparison of Quantitative Results with the Literature Data

We compared our results with those obtained using two other high-throughput methods (see Supplementary Table S2). We filtered the protein list to include only those proteins identified at a biotinylated site and not originating from contaminants in the cell culture medium, and assigned an exact apical percentage based on this study. We then compared the apical percentage of the filtered protein list to that of Caceres et al. and Koetemann et al. (only considering the apical percentages determined by both us and them). The correlation coefficients were positive in both cases: 0.48 and 0.61, respectively. These coefficients improved further when we used only the labeled TMPs from these protein sets: 0.67 and 0.63, respectively. Considering that the calculated apical percentages are based on different methods (such as labeled protein-level or peptide-level enrichment) in different laboratories, these correlations are satisfactory. Qualitative conclusions are made on proteins with a significant level of polarization; therefore, the correlation of those values with the dataset most similar in methodology (Koetemann et al.) is shown in Supplementary Figure S6. Apical % data of the matching proteins show an even better correlation (r = 0.74), with only 4% of proteins with opposite significant polarization.

## 3. Discussion

In our previous works on protein topology [30,39,40], the main task was to identify as many real extracellular biotinylated sites of CSPs as possible. However, the quantitative aspects of this process were not studied. In this work, we have developed methods to further filter real positive CSPs, evaluate the quantitative precision of the analytical methods, and select quality control parameters.

All extracellular labeling methods can enrich every protein that can be identified extracellularly. These include the targets of our analysis, CSPs, and the secreted proteins of the studied cells. In addition to these, proteins that remain after incomplete removal of cell growth media, as well as intracellular proteins released from damaged cells, are also found in the extracellular space. If the labeling agent can pass through the cell membrane during the entire process, biotinylation of additional intracellular proteins or intracellular segments of plasma membrane-bound proteins may occur. Non-biotinylated proteins/peptides binding non-specifically to the resin used for enrichment adds another source of false positives to the data. Identifying and classifying these different classes of “contaminants” is a complex but crucial task in the data analysis workflow and may also help improve sample processing protocols.

The most reliable approach to confirm protein biotinylation is detecting labeled peptides. The most effective method for identifying and quantifying such peptides is to enrich biotinylated peptides after protein digestion, as detailed in our previous works. However, the applicability of such data in quantitative analysis must be confirmed. The only comprehensive quantitative proteomics study to deal with the enrichment and analysis of peptide-level cell surface biotinylation was performed by Garapati et al. [41]. In their study, they applied an antibody to enrich biotinylated peptides, which was thought to facilitate the release and detection of these peptides compared to methods applying a stronger binder such as avidin/streptavidin [42]. In our method, we applied a disulfide-linked biotin, which enables the release of peptides without breaking the biotin–avidin bond. Thus, complete release of biotinylated peptides can be expected. Another advantage of our method is that it requires almost two orders of magnitude less sample (50–100 µg total protein in our study versus 6 mg in the sBioSITe method), enabling the quantitative analysis of single plates/transwells.

The application of the DIA data collection method for quantitative proteomics analysis promises increased proteome analysis depth, a lower number of missing values, and more reproducible quantitative data than the more traditional DDA mode [43]. However, data collection parameters (e.g., monitored mass over charge range) and analysis parameters (e.g., applied software and spectral library with appropriate chemical modifications included) need optimization for labeled and enriched peptides/proteins. DIA data analysis is generally based on spectral libraries, which can be obtained experimentally for a specific sample type or created in silico using different spectrum prediction algorithms. Until recently, spectrum prediction and DIA data analysis had limited applicability to chemically or post-translationally modified peptides, particularly when using large spectral libraries. Therefore, optimal library creation is crucial for in-depth proteome quantification. Gou et al. [44] applied both DDA and DIA modes to acquire data for analysis of biotinylated CSPs. However, they used DDA-based protein identification software on pseudo-MS/MS peak lists generated from the DIA data. Therefore, the results of their DIA data do not represent the real benefits and challenges of a completely DIA-specific approach [45].

In quantitative proteomics, filtering samples based on appropriate quality control (QC) measures is essential to obtain meaningful data, particularly when complex sample processing steps are employed, such as cell surface labeling and enrichment. Identified protein lists must be filtered to remove false positives and identify the most probable true positive cell surface/secreted proteins in order to draw accurate conclusions and assignments regarding cell surface polarization of specific proteins. Selecting marker proteins for both good-quality cell surface enrichment and different classes of possible contaminants can help to achieve this. In their previously mentioned work, Gou et al. [44] also suggested a list of selected cell surface proteins for quality control. However, peptide-level biotinylation efficiency was not considered in the selection process, and only qualitative information (detectability) was used for comparisons throughout the work. If quantitative information is included (e.g., ratio of selected proteins or intensity proportion of protein groups), more precise QC thresholds can be selected. In addition to QC, an appropriate group of proteins may also serve the purpose of quantitative normalization. When selecting QC proteins for the quantitative study of cell polarization, the apical/basolateral localization of specific proteins should be taken into account. However, the polarization of QC/normalization proteins may introduce bias into the quantitative data if the apical/basolateral surface ratio varies, even in global surfaceome analysis. Conversely, selecting negative QC proteins (contaminants) can facilitate the identification of suitable samples, particularly if a sample-specific contaminant database is established. Identifying contaminant proteins is also vital for quantitative normalization. Highly abundant contaminants may alter the total, median, and other protein intensities of samples, thus introducing bias into the quantitative data if traditional normalization approaches are used without removing contaminants from the dataset.

To validate our method, we performed polarization experiments in transwells with MDCKII dog cells. This is because the apical/basolateral topology of these cells has already been studied by others using proteomics methods [29,38].

### 3.1. Comparison of DDA and DIA Methods

In summary, we can expect more identifications and less missing data when analyzing a whole proteome with DIA, provided there are few variable modifications. However, during biotin enrichment, most of the unmodified peptides are removed from the sample and fewer peptides remain, most of which are chemically modified. In our experience, this may cause difficulties in calculating the false discovery rate (FDR) during DIA analysis, resulting in a higher rate of false discoveries or fewer identifications below the calculated FDR limit. Our current results confirm that the total number of identified peptides and proteins is higher when using DIA data with predicted spectral libraries in whole-sample lysates or protein-enriched samples, but lower when peptide-level enrichment was applied. In all cases, however, the number of identified biotin-labeled peptides is higher using DDA. To check the effect of the spectral library on the number of DIA identifications, we analyzed the same raw DIA data with an experimental library created from the DDA identifications. Using this library, we observed a significantly higher number of biotinylated peptide identifications in the peptide-level enriched samples. A lower number of identifications in DIA data may be the consequence of the inappropriate prediction of retention times and/or MS/MS fragmentation in predicted libraries, or inappropriate estimation of FDR. We found an excellent correlation of predicted and experimental retention times, both for modified and unmodified peptides (Supplementary Figure S5A). The distribution of q-values is similar for modified and unmodified peptides in each dataset, but this distribution is different in the case of protein- and peptide-enriched samples, which may be a result of a lower number of true-positive identifications in the latter dataset (Supplementary Figure S5B). This is also supported by the fact that in the case of protein enrichment, a larger number of identifications of both unmodified and modified peptides were observed using DIA than with DDA. Further investigation or optimization of DIA for analysis of the lower-complexity peptide-enriched samples was out of the focus of our work; we have concluded that, for the in-depth analysis of chemically confirmed cell surface proteins using our experimental setup (protocols and instrumentation), the DDA method is more effective. This may, however, not be true for other datasets collected using other methods and instruments, and analyzed by different software tools.

### 3.2. Cell Surface Polarization of MDCKII Cells

We detected 108 out of 235 proteins, predominantly apical TMPs and secreted proteins. PODXL, for example, is a transmembrane sialomucin and a well-known apical marker protein that plays a role in lumen formation during polarized epithelial morphogenesis [46]. Mucin1 (MUC1) has been detected by 28 different biotinylated peptides, making this one of the largest labeled peptide hit lists with topologically correct labeling. This apical result was confirmed earlier by Mattila et al. [47]. Another result consistent with the literature data is that protein tyrosine phosphatases, such as PTPRJ, localize to this membrane domain [48,49]. We also found biotinylated transporter proteins apically on the MDCKII cell surface. For instance, we detected the NPC1-like intracellular cholesterol transporter 1 (NPC1L1) protein apically, which was earlier detected at the apical membrane surface and intracellularly in Caco-2 cells and the human small intestine, with appearance in the brush-border membrane, a crucial site for lipid absorption [50].

As in other studies, ACE2 has been detected with a high mean intensity value and apical localization. This protein is a homolog of angiotensin-converting enzyme (ACE), which is central to the renin–angiotensin system (RAS). ACE2 is abundant in the human kidney and heart, playing an important role in renal and cardiac function through its ability to hydrolyze angiotensin II [51]. ACE2 is the cellular receptor for SARS-CoV and is localized on the apical plasma membrane of polarized respiratory epithelial cells, mediating infection from the apical side of these cells [52].

As was previously demonstrated [53,54], the CEACAM1 protein has also been identified apically. It belongs to the carcinoembryonic antigen-related cell adhesion molecules (CEACAMs), which are a subgroup of the carcinoembryonic antigen (CEA) family of immunoglobulin-related proteins. Members of this family, including human CEACAM1, CEA, and CEACAM6, are found on various epithelial cell types and derived carcinomas. They are thought to influence the interaction between tumor cells and their stromal and immune cell counterparts [54].

Furthermore, we identified several TMPs or secreted proteins that were present in higher quantities in the basolateral membrane domain of MDCKII cells. For instance, the ADAM Metalloproteinase Domain 10 (ADAM10) protein was quantified in the highest median level in this group of proteins. It plays an important role in adherent junctions, E-cadherin processing, and the migration of the polarized epithelial cells [55]. Our identification is cross-validated by the fact that the ADAM10 protein has been immunostained in the basolateral domain of polarized MDCKII cells, where it was found to have a sorting signal that directs it to this domain [55]. Its potential interacting partners were biotinylated extracellularly several times, such as CD44 antigen (CD44) [56] and E-cadherin [57]. In this study, it was mostly identified in the expected membrane region. CD44 was slightly more abundant, while the cadherin-1 and other cadherins (e.g., cadherin-6, cadherin-16) were presented in higher amounts in the basolateral domain than in the apical domain. These results are in accordance with cadherin knockdown experiments [58] and airway epithelial cell studies [59]. We found that some receptors, such as the receptor protein-tyrosine kinase (DDR1), the LDL receptor (LRP1), and the lipolysis-stimulated lipoprotein receptor (LSR), are enriched in this domain. The locations of these receptors have already been detected basolaterally by others too [29,60]. The ATP1B1 protein, which is essential for maintaining various epithelial barriers, was significantly detected in this domain by 13 significant basolateral peptides (out of the 51 detected peptides; see Supplementary Table S2 and Supplementary Figure S1), which is consistent with previous findings (e.g., yellow fluorescent protein-linked ATP1B1 has been detected in basolateral domains by surface-selective biotinylation followed by Western blot analysis [61]).

### 3.3. Comparison to Other Results

While the correlation of common proteins identified by Caceres et al. and Koetemann et al. was acceptable (see Section 2.5), the SILAC-based methods also produced contradictory results for some proteins compared to our own findings. Caceres et al. identified cadherin-6 and laminin subunit alpha 3 (LAMA3) in the apical membrane domains, whereas our results show that they are predominantly basolateral. These results are supported by two previous studies in which cadherin-6 was found on the basolateral surface [58] and LAMA3 was identified in the airway basement membrane [62]. Furthermore, Koetemann’s work also indicates that both are localized on the basolateral surface of the polarized MDCKII cells. A similar situation applies to heparan sulfate proteoglycan 2, an important building block of the extracellular matrix. Our method detected it in the appropriate membrane region (the basolateral membrane), which is in accordance with a previous study [63]. Thanks to biotinylated peptide enrichment, we also detected some apical proteins that were not identified in the work of Caceres et al. or Koetemann et al., for example, leishmanolysin-like peptidase or melanotransferrin, which is highly expressed on the apical surface of melanoma cells [64].

Using the described strict statistical methods, 235 significantly polarized proteins were identified. However, a large number of additional proteins were found to exhibit similar polarization tendencies to the core proteins. Therefore, to extend the range of potentially polarized proteins, we performed covariance analysis. Three apical and four basolateral protein clusters were identified, enriched with members of the polarized core, with strong intracluster correlation (r > 0.9). These polarized clusters include 450 proteins, which aligns well with the literature data and PolarProtDb database (see Table 1). We generally observed less than 10% false classification, except for a larger fraction of proteins with an apical classification in the Caceres et al. publication.

### 3.4. Interacting Protein Clusters Can Help Apical–Basolateral Annotation of Proteins

**Figure 5:**
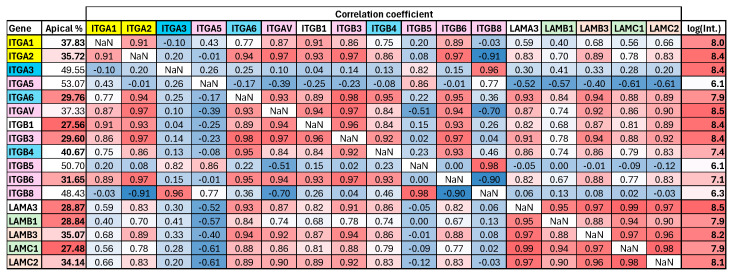
Covariance analysis of integrin (ITGA1-ITGB8) and laminin (LAMA3-LAMC2) subunits. Color codes for genes: light red—laminin-5 (laminin-332 or epiligrin/kalinin/nicein); light green—laminin-6 (laminin-311 or K-laminin) and laminin-7 (laminin-321 or KS-laminin); blue—laminin binding ITG; pink—RGD receptor ITG; yellow—collagen receptor ITG. High/low values are shaded ib red/blue, respectively. log(Int.) is logarithm of average MS intensity.

In all previous publications, individual protein entries were discussed. However, a large proportion of proteins occur on the cell surface in complexes consisting of several subunits or interacting with other proteins. Analyzing the covariance of these interacting protein chains may help to differentiate between polarized protein complexes with various compositions and confirm data on individual proteins occurring in the same complex. Integrins and laminins, which were previously selected for sample processing quality control, are also good models for analyzing cell surface complexes. Extracellular laminins consist of three subunits connected by disulfide bridges [62,65], while integrins are TMPs formed by the non-covalent interaction of two subunits. Complexes formed by the combination of different integrin subunits can serve as receptors for various proteins or protein classes, including fibrinogen and laminins [66,67,68]. Consequently, the colocalization of non-specific laminins with specific integrins can also be anticipated. Our peptide-based cell surface enrichment method is especially effective in analyzing complexes formed by disulfide bridges, as peptide enrichment occurs before reduction in disulfides. We observed only two laminins: laminin-311 and laminin-332. All subunits exhibited significant basolateral polarization, with distinct levels of polarization specific to the complexes. Specific members of Laminin-311 exhibit highly similar levels of polarization: 28.8% and 27.5% for the apical fraction for LAMB1 and LAMC1, respectively. Members of laminin-332 have a higher apical fraction—35.1% and 34.1% for LAMB3 and LAMC2, respectively. LAMA3 is present in both laminin forms. The observed high correlation coefficients are also consistent with this. ITGA3 + ITGB4 and ITGA6 + ITGB4 bind laminins, while the other integrin subunits detected are present in either RGD sequence receptors (e.g., in fibrinogen) or collagen receptors. ITGA3 was found to be non-polarized and negatively correlated with laminins. In contrast, ITGB4 and ITGA6 are similarly basolaterally polarized to laminins and both have stronger correlations with members of the laminin-332 complex. The collagen receptor subunits (ITGA1 and ITGA2) are slightly basolateral, while the RGD receptor subunits are not polarized at all or are only polarized to a slight extent (just below 40% apical fraction). Corralation of Integrins and laminins are shown on Figure 5.

In addition, 740 biotinylated proteins were detected in the labeled peptide-level-enriched samples. In other side-selective biotinylation-based studies, these modifications were either not searched for [29] or were searched for but the results were not presented [38]. Of the 740 biotinylated proteins, 533 have at least one extracellular labeled site (indicated by ‘OK’ in the ‘Biotin Topology Check’ column in the Supplementary Table S2), which strengthens the selectivity of the developed method for surface proteins. Filtering the list based on the UniTmp database [69] for labeled TM proteins, for which this method was specified earlier [30,39,40], 260 TMPs were identified with at least one labeled position. More than 81% of these had only extracellular labeled positions (211/260), while the remainder likely originated from damaged cells (as most originated from organelles such as the endoplasmic reticulum or mitochondrial membranes). According to the TMPs labeled peptides, 1835 biotinylated TMP peptides were detected. Of these, 94% were identified in the extracellular parts of TMPs in this work, confirming the high cell surface specificity of biotinylation.

In summary, this optimized, controlled method for polarized cells enables the quantification of the apical-to-basolateral distribution ratios for hundreds of CSPs and facilitates the identification of thousands of potential epitopes accessible extracellularly or on the cell surface. These protein segments could be valuable targets for clinical diagnostics, particularly in diseases where cellular polarization is disrupted. Apical proteins, for example, may become mislocalized to the basolateral domain and their extracellular fragments can be released into the bloodstream, where they can be detected using epitope-specific antibodies in patient blood samples.

Furthermore, these extracellular regions are promising targets for small-molecule inhibitors. The efficacy of such compounds can be evaluated using the same type of polarized cell monolayers described in this study. This allows changes in surface protein abundance across membrane domains and differences in transport kinetics to be assessed.

Extending this methodology to other monolayer-forming cell types, such as intestinal epithelial cells or cells that form the blood–brain barrier, makes it possible to identify additional apical or basolateral surface proteins. These findings could make significant contributions to various areas of biomedical research.

## 4. Materials and Methods

### 4.1. Cell Culture

MDCKII cells (CRL-2936) were obtained from the American Type Culture Collection (ATCC, Manassas, VA, USA). The cells were grown in complete medium (CM)—Dulbecco’s modified Eagle medium (DMEM, Thermo Fisher Scientific, Waltham, MA, USA), containing 10% FBS (Gibco, Thermo Fisher Scientific) and 50 µg/mL penicillin–streptomycin (Gibco, Thermo Fisher Scientific)—inside a humidified incubator with 5% CO_2_ at 37 °C (Eppendorf, Hamburg, Germany; Galaxy 170R). Cells were passaged every 2–3 days under a laminar box (ESCO Class II Bsc).

Before the side-selective labeling, the MDCKII cells were grown in transwell inserts (using a 6-well plate: the surface area of the insert is 4.67 cm^2^ and the insert contains a 0.4 μm pore membrane, VWR) for six days. The mentioned culture time was determined by transepithelial electrical resistance/TEER measurements, when the electrical resistance of the cell layer was determined. This method can be applied to quantify the barrier integrity of cells (during their growth) [70,71].

### 4.2. Transepithelial Electrical Resistance (TEER) Measurements

For TEER measurements, polycarbonate transwell inserts (A = 0.6 cm^2^ area, 0.4 µm pore size, Millicell (R) Standing Cell Culture Inserts, PIHP01250, Merck Millipore Ltd., Burlington, MA, USA) were placed in 12-well plates (Sarstedt, Nümbrecht, Germany). Each insert was filled with 450 µL medium on the apical side and 1800 µL medium in the basolateral compartment, volumes chosen to avoid hydrostatic pressure differences and to submerge the TEER electrodes. Inserts were equilibrated with CM for at least 2 h before seeding. To establish a barrier layer, 40,000 MDCKII cells were seeded into the apical chamber. Transwell barrier cultures and cell-free (blank) inserts were maintained up to 15 days under standard culture conditions in CM and monitored daily by TEER measurements.

Transepithelial electrical resistance (TEER) was measured using TEERScanner, a prototype automated scanning system (BioPhys-Concepts Kft, Budapest, Hungary, biophys-concepts.com) equipped with MERSSTX01 chopstick electrodes (Merck, Darmstadt, Germany) connected to a Millicell ERS-2 voltohmmeter (Merck, Darmstadt, Germany). During measurements, the 12-well plate was placed on a heated stage to maintain a constant temperature of 37 °C, and transwell inserts were positioned within the wells using custom positioners to ensure consistent electrode placement.

Each measurement session involved two full scans of both the cell-free (blank) inserts and the inserts containing epithelial barriers. The entire scanning process for a 12-well plate was completed within approximately 5 min. In each scan, the TEER value of culture i was calculated as

TEER_i_ = [R_i_ − R_ref_] A, where R_i_ is the measured electrical resistance of culture i and R_ref_ is the average resistance of the blank inserts.

### 4.3. Cell Surface and Apical/Basolateral Surface Biotinylation and Its Control Isolates

The MDCKII cell monolayer was confluent after six days; therefore, we chose this culture time to label the polarized cells (TEER measurements can be seen in Supplementary Figure S2). First, the used medium was carefully removed, and the cells were washed three times with phosphate-buffered saline (PBS) solution (the components can be seen in [39]). Then, the adherent cells were washed once with PBS (pH 8.0) and labeled with 2 mM Sulfo-NHS-SS-biotin (Thermo Fisher Scientific) in a HPMI buffer (see in [13,72]) at room temperature for 10 min. The shorter labeling time period (relative to our previous works) was required in the case of MDCKII cell monolayers to avoid damage to the cell monolayer and to minimize the transfer of the labeling agent to the opposite side of the cell monolayer (to avoid the opposite cell surface labeling, a HPMI solution was used containing 2 mg/mL bovine serum albumin (BSA) on the opposite side of the inserts under the labeling time). The reaction was stopped by Tris-buffered saline (TBS) solution (see [39]); the TBS wash was repeated three times. The labeled cells were harvested from the transwell inserts using cell scrapers.

The culture media-treated flasks (without cells) were washed, labeled, and scraped similarly; that was the first control sample (for the identification of the non-specific protein pool that originated from the culture medium). The protein content of the culture medium was concentrated using 3 kDa Amicon Ultra Centrifugal filters (UFC900324, Merck Millipore) at 3000× *g* for 30 min at 4 °C. The concentrated sample was used as another protein background control (unlabeled/labeled control samples were prepared from this isolate).

### 4.4. Labeled Cell Lysis and Membrane Preparation

The scraped cells were incubated in ice-cold hypotonic lysis buffer (20 mM Tris–HCl, 10 mM KCl, 20 mM sucrose, 10 mM iodoacetamide (IA), pH 7.4) for 10 min at 4 °C. The further membrane preparation process was similar to that described in our previous works [30,39,73].

The white membrane pellet was homogenized by 25 strokes with a Potter–Elvehjem PTFE pestle in a glass tube (2 mL, Sigma-Aldrich, St. Louis, MO, USA) in the diluted lysis buffer on ice. The membrane preparations were stored at −20 °C until further use. The protein content of the labeled membrane isolate was measured using the Lowry method [74] using a BSA protein dilution series.

### 4.5. Labeled Peptide/Protein Enrichment Sample Preparation Protocol from Isolated Membranes

The apical/basolateral surface (MDCKII) labeled membrane preparations (with 50–100 µg protein content based on Lowry measurement) were solubilized by 0.1% (m/V) RapiGest SF (Waters) in 50 mM ammonium-bicarbonate (AmBic), using a chilled bath sonicator (Joan Lab Equipment Co., Ltd., Huzhou, China) in a cold room (5 × 1 min consecutive sonication). The solutions were incubated on ice for 30 min (gently vortexed every 5 min), and to improve the membrane protein solubilization, the samples were boiled in a dry-block thermostat for 5 min at 95 °C. After the solutions were put back on ice, IA and 2,2′-Thiodiethanol were added in 1.25–1.25 mM final concentrations and incubated at 37 °C for 30 min in the dark, after which, the protein-level enrichment followed. In the case of the labeled peptide enrichment protocol, the labeled membrane preparations were treated as described in our previous works [30,40].

The labeled peptides/proteins were enriched on high-capacity neutravidin agarose resin (Thermo Fisher Scientific). The optimal amount of resin was determined by the dot blot method (as described in our previous work [39]) and was then loaded into a Pierce Snap Cap Spin-Column; the labeled peptides/proteins were isolated at room temperature for 60 min. Thereafter, the resin was washed extensively as described previously [30]. The enriched peptides/proteins were eluted with 10 mM dithiothreitol (DTT, Thermo Fisher Scientific) reducing agent in 50 mM AmBic buffer for 30 min at 37 °C (this step was repeated once). The eluted peptides/proteins were alkylated with 25 mM IA in the dark at 37 °C for 30 min.

In the case of the protein-level enrichment protocol, two different methods were used; the first was the on-pellet digestion, after acetone precipitation (the short name is Protein–OP), and the second was the filter-aided digestion method (hereinafter referred to as Protein–FA). In the case of Protein–OP, 7x the volume of pre-cooled acetone was added to the samples and the protein pellets were left to precipitate overnight at −20 °C. The next morning, the precipitates were centrifuged at 14,000× *g* for 10 min at 4 °C, and the pellets were washed twice with an 85:15% acetone/water mixture. The protein pellets were resolved in 0.1% (m/V) RapiGest SF in 50 mM AmBic, and protein contents were determined using the Lowry assay [74]. Samples were then treated with PNGaseF (for 2 h at 37 °C, 250 units/sample) and digested with proteomics-grade trypsin (1:25 (*w*/*w*) enzyme/protein ratio) and incubated overnight at 37 °C, similarly to in the case of the peptide-level avidin enrichment. The next day, the reactions were stopped by formic acid. In the case of the Protein–FA protocol, the eluted protein solutions were placed into a 10 kDa membrane filter (Millipore Sigma Microcon-10 kDa Centrifugal Filter Unit with Ultracel-10 membrane), where the proteins were denatured with 8 M urea in 50 mM AmBic buffer, and similarly alkylated with 25 mM IA. The buffer was changed to 50 mM Tris-HCl (pH 8.5) and the proteins were digested overnight at 37 °C on the filter. The next day, the filters were centrifuged at 10,000× *g* at 20 °C for 20 min, and the peptides containing flow-through fractions were collected.

The peptide mixtures were dried using a SpeedVac concentrator in all cases. The previously optimized C18 purification [30] was made and stored at −20 °C until the mass spectrometry analysis.

### 4.6. LC-MS Analysis

All DDA and DIA LC-MS measurements were performed on a Waters ACQUITY UPLC M-Class LC system (Waters, Milford, MA, USA) coupled with an Orbitrap Exploris 240 mass spectrometer (Thermo Fisher Scientific, Waltham, MA, USA). A symmetry C18 (100 Å, 5 µm, 180 µm × 20 mm) trap column was used for trapping and desalting the samples. The chromatographic separation of peptides was accomplished on an ACQUITY UPLC M-Class Peptide BEH C18 analytical column (130 Å, 1.7 µm, 75 µm × 250 mm) at 45 °C by gradient elution. Water (solvent A) and acetonitrile (solvent B), both containing 0.1% formic acid, were used as mobile phases at a flow rate of 200 nL/min. The sample temperature was maintained at 5 °C. The mass spectrometer was operated using the equipped Nanospray Flex Ion Source. DDA measurements were collected using a method with an MS1 scan between 360 and 2200 Th using 60,000 resolution, while ddMS2 scans with isolation windows of 2 Th were collected at 30,000 resolution, keeping a 3 s cycle time. Data acquisition was performed using XcaliburTM 4.6 (Thermo Fisher Scientific, Waltham, MA, USA). Raw LC-MS data files were processed using Fragpipe v22.0 [75]. A dog (43,622 proteins) reference proteome, assuming two missed cleavage sites, was used for protein identification. An initial open search (using default settings) was performed to identify detectable peptide modifications. Based on this, the final quantitative database searches were performed assuming modifications listed in Supplementary Table S3. Protein quantification was performed within Fragpipe using IonQuant with default settings and enabling MBRs. A contaminant database was created using a database search of LC-MS data collected from a labeled and enriched digest of cell medium using the same search settings and the Uniprot Bovine reference proteome database (26,942 proteins). Based on analysis of cell growth medium, a contaminant database including 290 bovine proteins, porcine trypsin (TRYP_PIG), and neutravidin (AVID_CHICK) was built. Proteins were identified with bovine-specific peptides.

DIA measurements were collected on a m/z range from 380 to 1220 Th, using a cycle time of 3 s. Data analysis was performed using DIA-NN version 2.02 [76]. MBR, protein inference, and peptidoform scoring were enabled. Three spectral libraries were evaluated for identification with different sizes and sources of spectra, as these may largely affect the identification rate for small datasets [77,78]:

(a) Library created by Fragpipe (EasyPQP module) from experimental DDA measurements (DDA library) (22,106 precursors);
(b) Library predicted by DIA-NN from the same sequence database as used for DDA (predicted library using proteome) (28,561 precursors);
(c) Library predicted by DIA-NN from sequence database containing protein sequences identified in DDA measurements (predicted library using DDA proteins) (5,893,117 precursors).

Statistical analysis of proteomics data was performed in Perseus 1.6.15 [79] and Instantclue v0.12.2 [80].

Replicate average-based normalized intensity in MDCKII samples for apical/basolateral comparison was calculated using the following equation for each protein:

(1) $$I_{normalized}(replicate,side)=\frac{I(replicate,side)}{I(replicate,average)}\times I(total,average)$$

where *I(replicate, side)* is the measured intensity on the specific side (e.g., *I_(1_1, apical)_, I_(1_1, basolateral)_*) of the given replicate; *I(replicate, average)* is the average intensity of the two sides of the given replicate; and *I(total, average)* is the average intensity of all samples. A graphical representation of this approach is shown in Supplementary Figure S3. The method assumes similar variations in intensity of both sides of biological replicates related to total protein expression and sample processing (labeling, extraction, enrichment). It also assumes that these variations appear in the average abundance measured in each replicate. The method was applied to both peptide- and protein-level data before differential expression analysis. Normalization comparisons were performed using limma [81] and ComBat [82] batch correction methods, defining bioreplicates as batches.

## Figures and Tables

**Table 1:** Comparison of the polarized protein clusters identified based on covariance, with the literature data [29,38] and PolarProtDb database. Column Significant, TopoOK, and TM show the number of proteins with significant peptides, the number of proteins where all labeled peptides were in the topological right position (i.e., extracellular), and the number of transmembrane proteins, respectively. In the case of Caceres et al.’s publication, the total number of polarized proteins and significantly polarized proteins are also shown; the latter is shown in parentheses. The “Total” row indicates the number of those proteins that were detected with at least one strong correlation (r > 0.95) based on the covariance analysis (without contaminant proteins).

					Apical			Basolateral		
Cluster	Proteins	Significant	TopoOK	TM	Koetemann	Caceres	PolarProtDb	Koetemann	Caceres	PolarProtDb
Apical	221	96	114	47	21	73 (31)	13	6	6 (2)	1
Basolateral	229	112	140	86	2	50 (18)	2	56	53 (39)	18
Total	1481	235	453	305	35	454 (199)	23	120	132 (85)	40

## Data Availability

The raw LC-MS files of peptide-enriched samples and quantitative results from Fragpipe analysis are available from the PXD071272 ProteomExchange identifier.

## Supplementary Materials

The following supporting information can be downloaded at https://www.mdpi.com/article/10.3390/ijms262311570/s1.

## Abbreviations

The following abbreviations are used in this manuscript:

ACE	Angiotensin-I converting enzyme
ACE2	Angiotensin-converting enzyme-2
ADAM9	A disintegrin and metalloproteinase domain-containing protein 9
ADAM10	A disintegrin and metalloproteinase domain-containing protein 10
AmBic	Ammonium-bicarbonate
ATCC	American cell type culture collection
ATP1B1	ATPase Na+/K+-transporting subunit beta 1
BSA	Bovine serum albumin
CD44	CD44 antigen
CEA	Carcinoembryonic antigen
CEACAM1	Carcinoembryonic antigen-related cell adhesion molecule-1
CEACAM6	Carcinoembryonic antigen-related cell adhesion molecule 6
CM	Complete medium
CSP	Cell surface protein
DDA	Data-dependent acquisition
DDR1	Epithelial discoidin domain-containing receptor 1
DIA	Data-independent acquisition
DMEM	Dulbecco’s modified Eagle medium
DTT	Dithiothreitol
EYFP	Enhanced yellow fluorescent protein
FBS	Fetal bovine serum
FDR	False discovery rate
HPMI	Hepes-buffered medium RPMI 1640
IA	Iodoacetamide
ITGA1/2/3/6	Integrin subunit alpha 1/2/3/6
ITGB4/8	Integrin subunit beta 4 or 8
LAMA3	Laminin subunit alpha 3
LAMB1	Laminin subunit beta 1
LAMC1/2	Laminin subunit gamma 1 or 2
LC-MS	Liquid chromatography–mass spectrometry
LFQ	Label-free quantification
LLC cells	Lewis lung carcinoma cells
LRP1	Low-density lipoprotein receptor-related protein 1
LSR	Lipolysis-stimulated lipoprotein receptor
MBR	Match between runs
MDCK	Madin–Darby canine kidney
MDR1	Multidrug resistance protein 1/P-glycoprotein
MUC1	Mucin1
NaPi-2b	Sodium/phosphate cotransporter 2B
NHS	N-hydroxy succinimide
NPC1	Niemann–Pick C1 protein
NPC1L1	NPC1-like intracellular cholesterol transporter 1 protein
PBS	Phosphate-buffered saline
PODXL	Podocalyxin
POI	Protein of interest
PTPRJ	Protein tyrosine phosphatase receptor type J
QC	Quality control
RAS	Renin–angiotensin system
RGD	Arginylglycylaspartic acid (sequence/motif)
sBioSITe	surface Biotinylation Site Identification Technology
SD	Standard deviation
SILAC	Stable isotope labeling by amino acids in cell culture
TBS	Tris-buffered saline
TEER	Transepithelial electrical resistance
TMP	Transmembrane protein
UniTmp	UNIfied database of TransMembrane Proteins
YFP	Yellow fluorescent protein
