# Supplementary material for: Optimization of Methods for the Quantitative Analysis of Global Cell Surface Proteome and Cell Surface Polarization
Source: Int J Mol Sci. 2025 Nov 28;26(23):11570. doi: 10.3390/ijms262311570 (PMC12692164; doi:10.3390/ijms262311570)
Supplement: Supplementary file 1 [file ijms-26-11570-s001.zip › Supplementary_Figures.pdf]

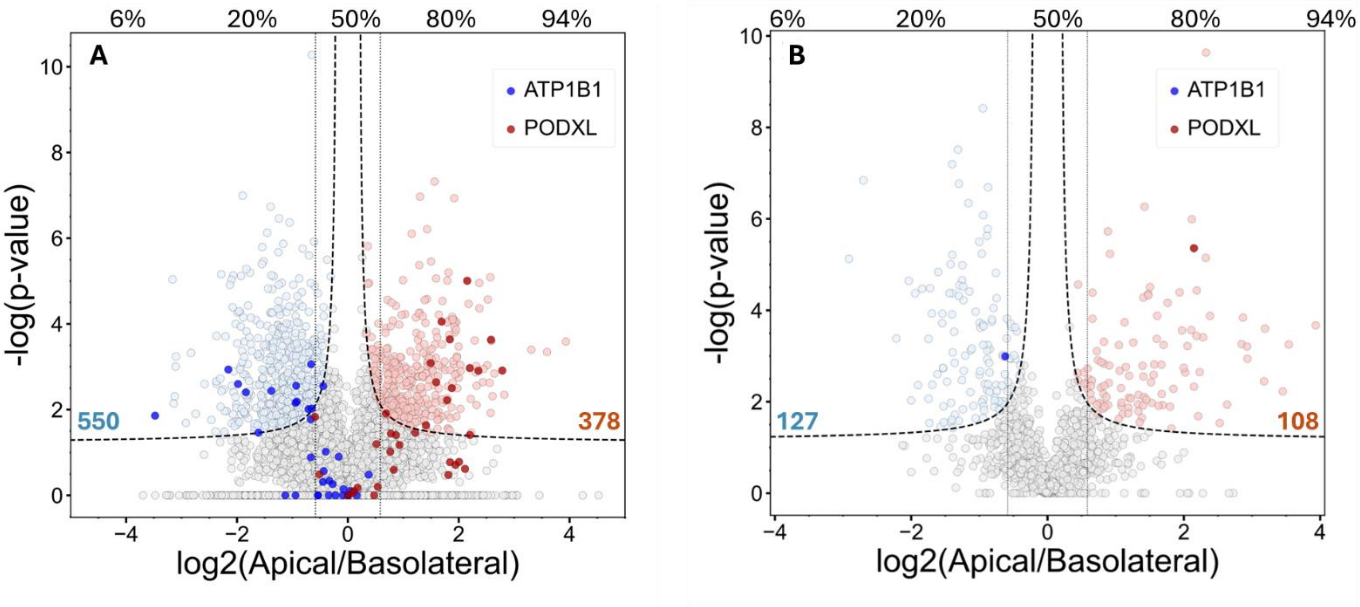

Supplementary Figure S1. Volcano plot representation of differential analysis at peptide (A) and protein (B) level. Statistically significant apical and basolateral polarization is shown by light red or blue color respectively. Data points of representative apically (PODXL) and basolaterally (ATP1B1) polarized proteins selected based on literature data are highlighted by dark colors. Total amount of significantly increased/decreased peptides/proteins are shown by red and blue numbers.

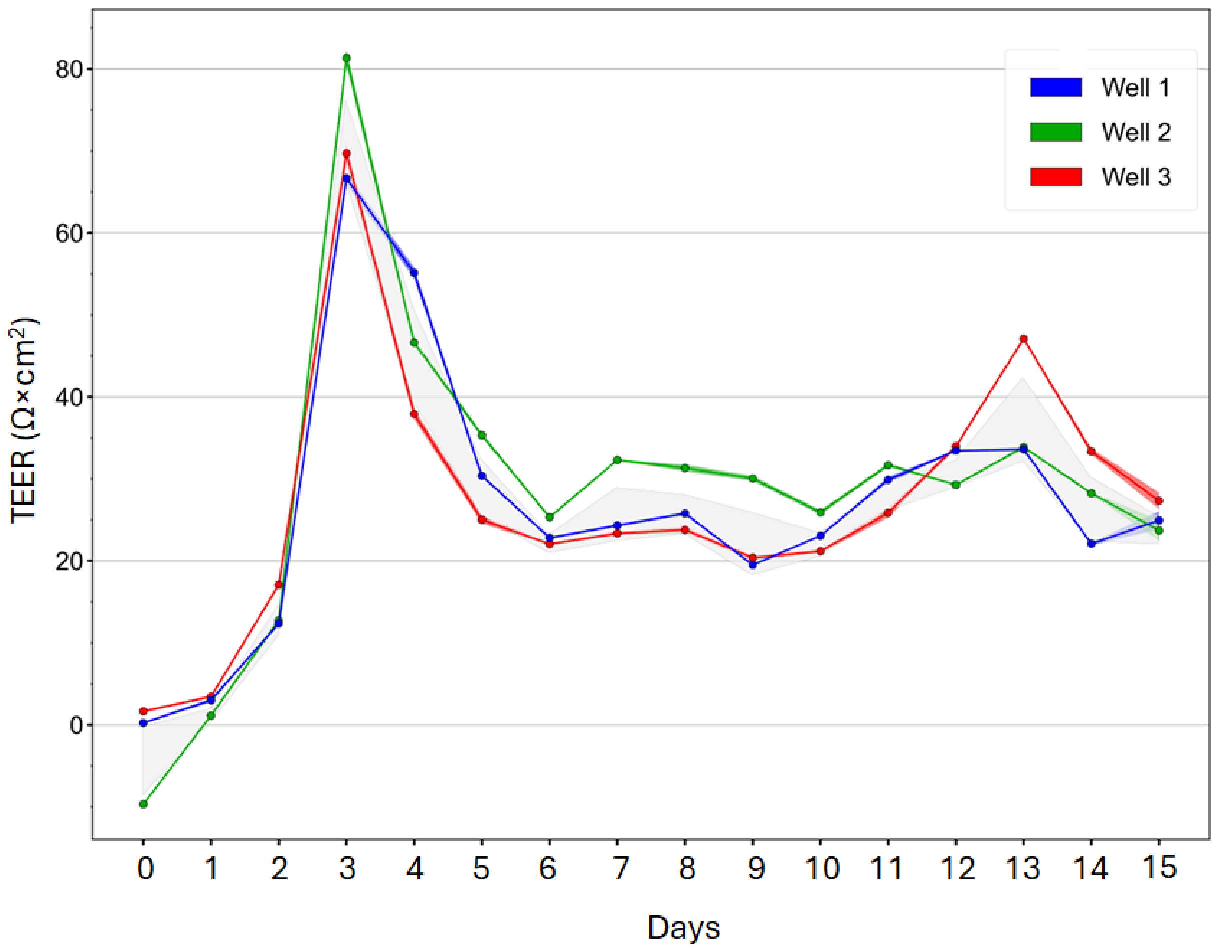

Supplementary Figure S2. Time course of TEER values in MDCKII barrier layers. MDCKII cells were seeded at a density of 35 × 10^3 cells/cm^2, and TEER was measured daily in transwell inserts. After a short lag phase with rising TEER values (Days 0–3), a transient decline occurred (Days 3–6), followed by a stable plateau phase (Days 6–15) when cultures were polarized and suitable for labeling experiments. TEER values from three parallel cultures are shown. Shaded areas indicate interquartile ranges for each culture (red, green, blue) and for the three cultures (grey).

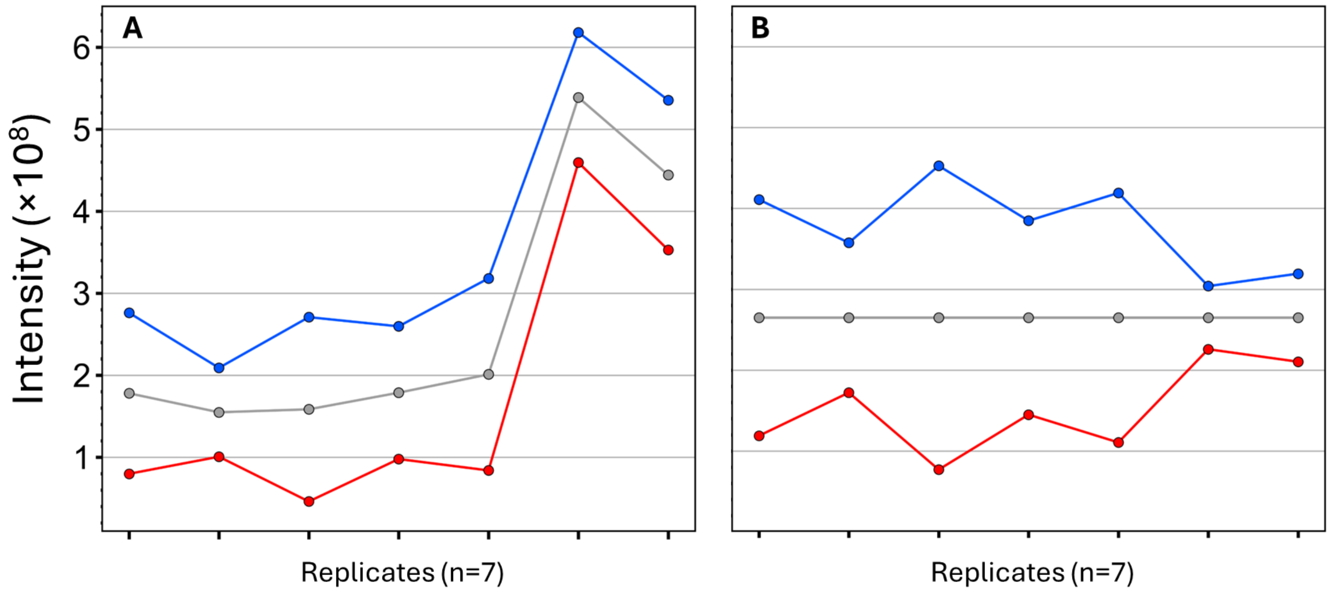

Supplementary Figure S3. Graphical representation of replicate-based normalization approach on the intensity data of ITGB1. Apical (red), basolateral (blue) and average (grey) intensities are shown before (A) and after (B) normalization using Equation (1).

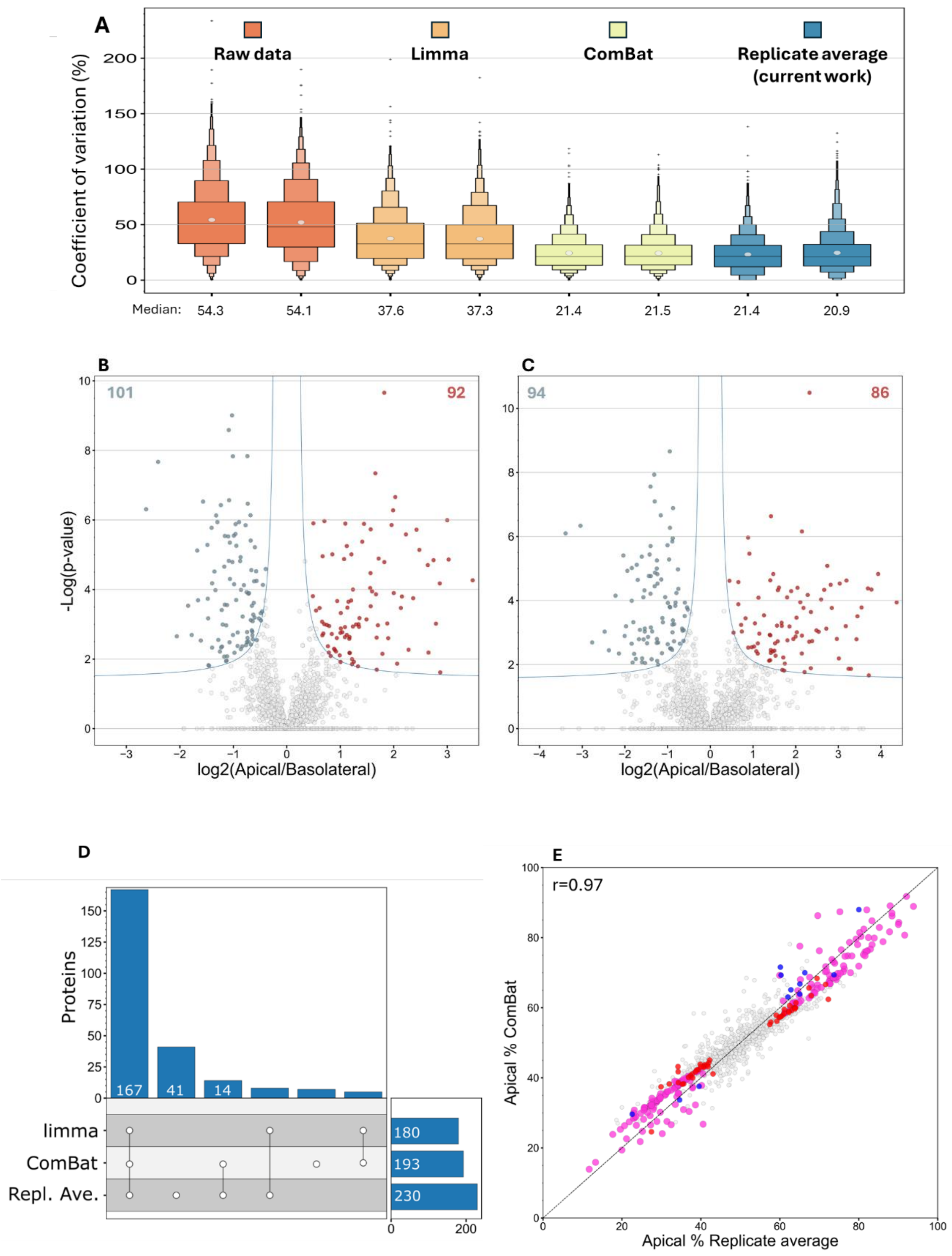

Supplementary Figure S4. A.: Distribution of coefficient of variance of raw data and after using different normalizations. Apical and basolateral data (n=7) are shown for each normalization (left and right, respectively). Median values are shown inside middle quantile box.

B. and C.: Volcano plots after different batch correction normalizations: A. ComBat B. limma algorithm. Biological replicates (apical/basolateral pairs) were defined as batch groups in each case. Total amount of significantly increased/decreased proteins are shown by red and blue numbers.

D. Number of significantly polarized proteins (FDR<0.05) found after different normalization methods.

E. Correlation of Apical % values calculated after ComBat and Replicate average-based methods. Points of proteins significantly different after only ComBat (blue), only replicate average based (red) and with both methods (violet) are colored.

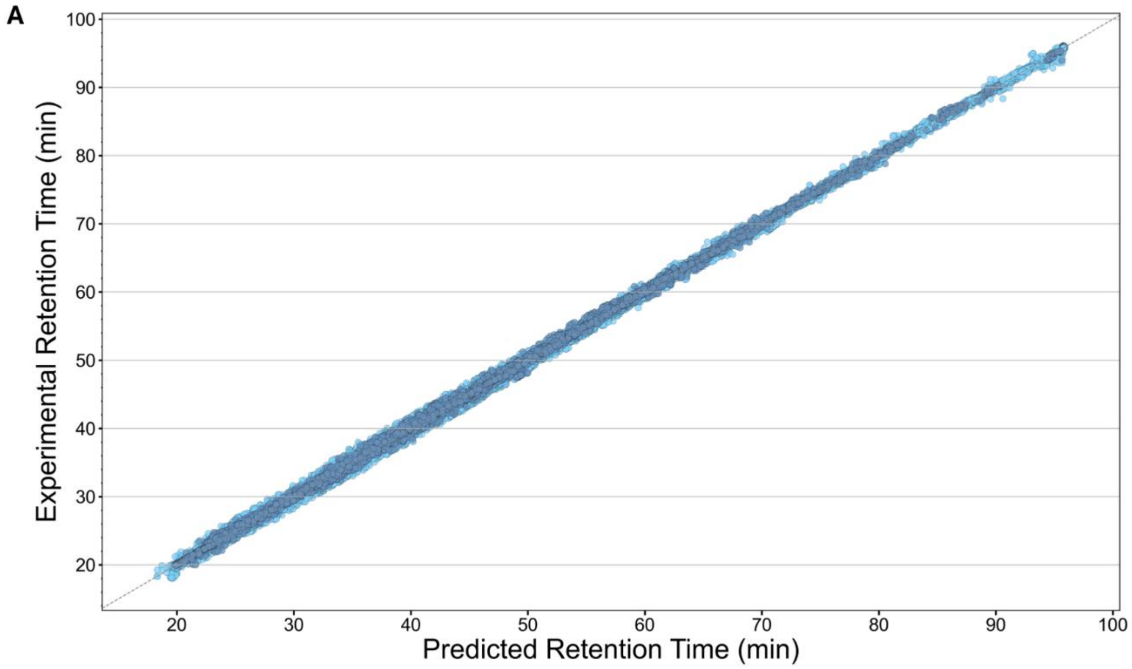

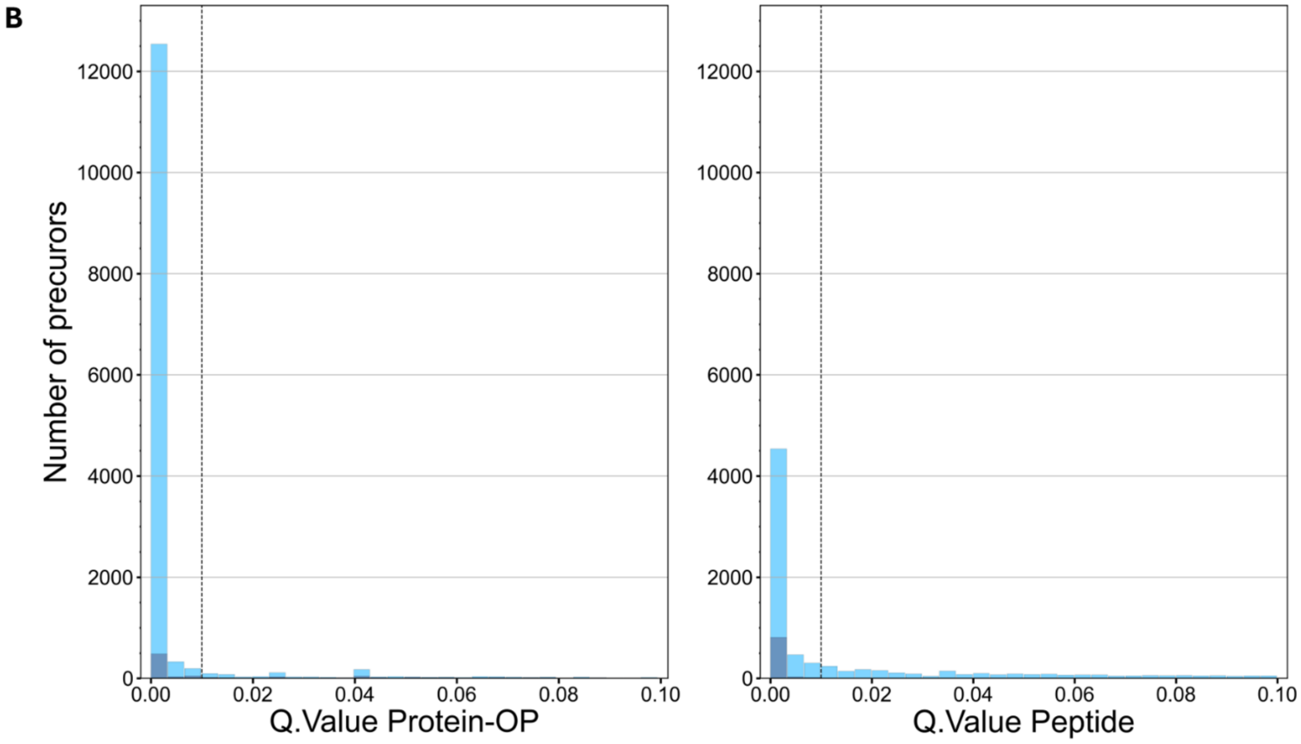

Supplementary Figure S5. A. Correlation of experimental retention times with values predicted by DIA-NN. All data identified points (q<0.01) of all samples are shown. Dark dots represent labeled precursor ions with Unimod:293 modification. B. Distribution of q values calculated by DIA-NN in datasets collected from protein enrichment with on pellet digestion (Protein OP) and from peptide enrichment (Peptide). Dark data represent labeled precursor ions with Unimod:293 modification Data for q values are shown between 0 and 0.1, significance threshold of q<0.01 is represented by vertical dashed line.

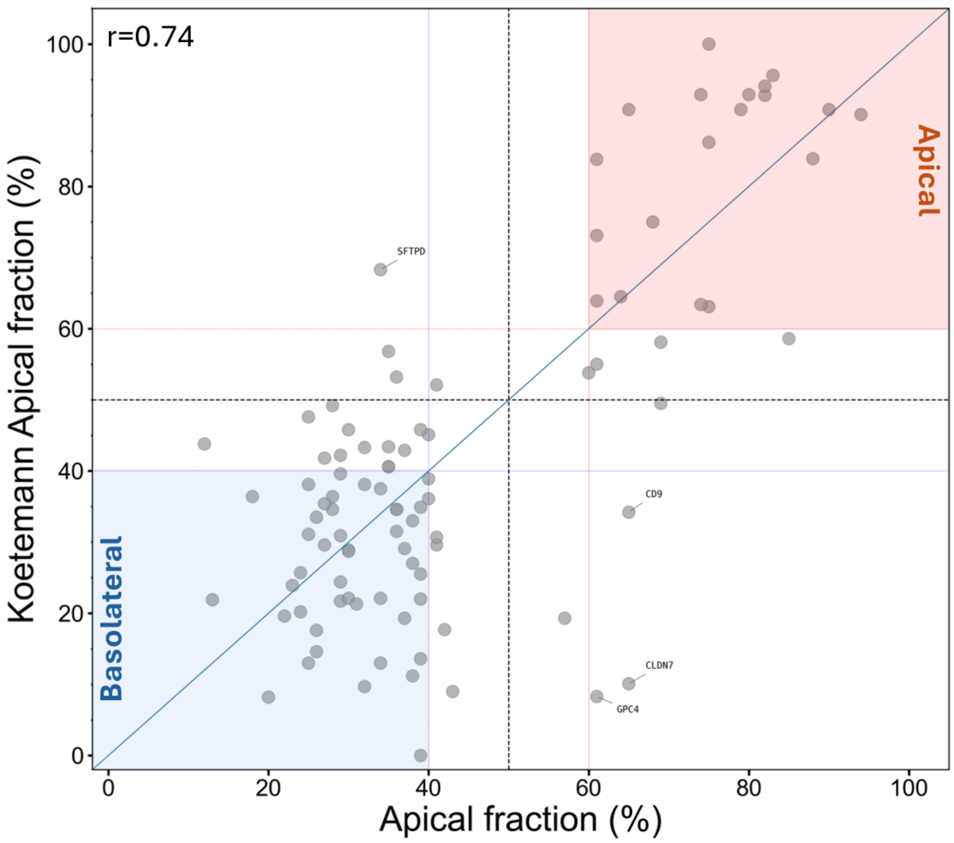

Supplementary Figure S6. Correlation of statistically significant quantitative polarization data from this work and Koetemann et al [38].
